# Cloning, expression and characterization of a versatile Baeyer-Villiger monooxygenase from *Dietzia* sp. D5

**DOI:** 10.1186/s13568-014-0023-1

**Published:** 2014-03-20

**Authors:** Serena Bisagni, Rajni Hatti-Kaul, Gashaw Mamo

**Affiliations:** 1 Department of Biotechnology, Center for Chemistry and Chemical Engineering, Lund University, Lund, SE-22100, Sweden

**Keywords:** Baeyer-Villiger monooxygenase, Biocatalysis, Enzyme stability, Protein expression

## Abstract

A novel BVMO encoding gene was identified from a draft genome sequence of a newly isolated strain of *Dietzia.* Analysis of the protein sequence revealed that it belongs to a group of BVMOs whose most characterized member is cyclopentadecanone monooxygenase (CPDMO). The gene was PCR amplified, cloned and successfully expressed in *E. coli*. The expressed recombinant enzyme was purified using metal affinity chromatography. Characterization of the purified enzyme revealed that it has a broad substrate scope and oxidized different compounds including substituted and unsubstituted alicyclic, bicyclic-, aliphatic-ketones, ketones with an aromatic moiety, and sulfides. The highest activities were measured for 2- and 3-methylcyclohexanone, phenylacetone, bicyclo-[3.2.0]-hept-2-en-6-one and menthone. The enzyme was optimally active at pH 7.5 and 35°C, a temperature at which its half-life was about 20 hours. The stability studies have shown that this enzyme is more stable than all other reported BVMOs except the phenylacetone monooxygenase from the thermophilic organism *Thermobifida fusca*.

## Introduction

Baeyer-Villiger reaction was discovered more than one hundred years ago and refers to the oxidation of a ketone to a lactone or an ester (Baeyer and Villiger [1899]). It is regarded as one of the important reactions in chemical industry and is currently accomplished in organic solvents using peroxyacids as oxidants. However, these compounds are harmful and explosive; hence it is considered that Baeyer-Villiger reaction using these oxidants is problematic and unsafe in large-scale industrial reactions (Stewart [1998]). Discovery of enzymes known as Baeyer-Villiger monooxygenases (BVMOs) which are able to catalyze such reactions has led to possible alternative of using them as safe and green catalysts for Baeyer-Villiger reactions. In line with this, in the last fifteen years, more than fifty BVMOs have been cloned and expressed in heterologous systems and a number of other BVMOs have also been directly purified from different wild type microorganisms and characterized (Leisch et al. [2011]).

BVMOs are flavin-dependent enzymes which require NADPH for regeneration of FAD and use molecular oxygen as the oxidizing agent. These enzymes are known to catalyze the oxidation of ketones and heteroatoms such as sulphur, nitrogen, boron, phosphorus and selenium (Walsh and Chen [1988]). In addition to safety, the use of BVMOs allows to run reactions with high enantio- and regio-specificity, which is often difficult to achieve when chemical catalysts are employed. In applications where the chiral nature of the product is an important factor such as in pharmaceutical industries (Pollard and Woodley [2007]; Ramesh [2008]), the use of BVMOs is of great interest. However, despite such advantages, the application of BVMOs in industrial processes (Baldwin et al. [2008]) is hampered due to lack of robust enzymes, complexity of the reaction processes, such as substrate and product inhibition, and difficult downstream processing. One of the major bottlenecks is the poor stability of BVMOs (Leisch et al. [2011]). With the exception of phenylacetone monooxygenase (PAMO) obtained from a strain of the thermophilic bacterium *Thermobifida fusca* (Fraaije et al. [2005]), all the other known BVMOs are not stable and very rapidly lose activity even at room temperature (Völker et al. [2008]; Rehdorf et al. [2009]; Kadow et al. [2012]). Moreover, the activity and stability of BVMOs are known to be affected by organic solvents (de Gonzalo et al. [2006]; Secundo et al. [2011]). More stable BVMOs can be potentially obtained by rigorous protein engineering work, and new enzyme discovery by means of conventional microbial screening or from metagenome libraries and genome sequences.

In this article, we describe the cloning, expression and characterization of a novel BVMO from a strain of *Dietzia,* an organism previously isolated from a soda lake water sample. Although a number of *Dietzia* were isolated and reported to have rich oxygenase diversity, there has been no BVMO isolated and characterized from these organisms.

## Materials and methods

### Chemicals

All chemicals were purchased from Sigma Aldrich (Stockholm, Sweden), Calbiochem (Darmstad, Germany) and VWR (Stockholm, Sweden). All the chemicals for PCR, T4 DNA ligase and FastDigest® restriction enzymes were purchased from Fermentas (St. Leon-Rot, Germany). QIAGEN Plasmid Mini Kit and QIAEX II Gel Extraction Kit were purchased from Qiagen (Sollentuna, Sweden).

### Microorganisms and plasmid

*Dietzia* sp. D5 isolated in our laboratory is deposited in Culture Collection, University of Göteborg (CCUG 64924), Sweden and its genomic DNA was purified using ZR Fungal/Bacterial DNA MiniPrep (Zymo Research, Irvine, USA). *E. coli* NovaBlue, BL21(DE3), Rosetta2(DE3) and the plasmids pET-22b(+) were purchased from Novagen (Darmstad, Germany). *E. coli* BL21-CodonPlus(DE3)-RP and ArcticExpress(DE3)-RP cells were purchased from Agilent Technologies (Santa Clara, USA).

### Gene analysis and cloning

The gene encoding the monooxygenase BVMO4 was identified from the draft genome sequence of *Dietzia* sp. D5 (unpublished data). Identification of ORFs and analysis of the BVMO4 gene and its deduced protein sequence was performed with CLCBio Main Workbench (Aarhus, Denmark) FGENESB (Soft Berry Mount Nysco, USA) and BLASTp at NCBI. The phylogenetic tree was generated using the software FigTree.

The BVMO4 encoding gene was amplified by PCR from *Dietzia* sp. D5 genomic DNA sample using a pair of primers, forward primer ATTACCATGGCCTTCACCCTCCCTG and reverse primer ATTAGCGGCCGCGGCCACCGGGACCGCGTCG which have the *Nco*I and *Not*I restriction sites, respectively. The PCR amplification was performed with High Fidelity PCR Enzyme Mix (Fermentas) following the manufacturer protocol but supplemented with 2.5% (v/v) DMSO. The PCR product was purified using PCR cleaning kit and digested with *Nco*I and *Not*I. The digested DNA was loaded on agarose gel and after electrophoresis it was extracted from the gel using Qiaex II gel extraction kit, and ligated to the expression plasmid pET-22b(+) which was digested using *Nco*I and *Not*I. The ligation mix was transformed into competent *E. coli* NovaBlue cells and transferred to ampicillin containing LB-agar plates. After overnight incubation colonies were screened by PCR and plasmids from the insert positive colonies were extracted, sequenced at GATC Biotech AG, Konstanz, Germany and plasmids with correct sequences were transformed to the expression hosts.

### Protein expression and purification

Recombinant *E. coli* BL21(DE3), Rosetta(DE3), ArcticExpress(DE3)-RP and BL21-CodonPlus(DE3)-RP cells were grown in LB medium containing, whenever required, ampicillin 100 μg/ml, chloramphenicol 34 μg/ml and gentamycin 20 μg/ml, respectively. The cultures were incubated at 30°C with shaking (180 rpm), until the OD_600_ reached 0.6, then induced with 1 mM IPTG. After overnight induction at 15°C, the cultures were harvested by centrifugation, resuspended in 20 mM sodium phosphate buffer, pH 7.4 and disrupted by sonication for three rounds of 45 sec burst at 50% amplitude and 50% cycle, and 1 min break. The cell homogenate was centrifuged at 4°C for 15 minutes at about 15000 *g* using a Sorvall centrifuge and the clear supernatant used as source of the recombinant enzyme.

The His-tagged recombinant enzyme was purified at 4°C by Ni-NTA affinity chromatography using HisTrap™ FF crude column (GE Healthcare, Uppsala, Sweden) following the manufacturer instructions. After elution from the column, the enzyme was desalted and concentrated using Vivaspin 20 MWCO 30,000 centrifugal concentrators (Sartorius Stedim Biotech GmbH, Goettingen, Germany), FAD was added to a final concentration of 10 μM and stored at 4°C in 50 mM sodium phosphate buffer, pH 7.5. The homogeneity of purified sample was checked using 10% SDS-PAGE prepared according to Laemmli (Laemmli [1970]).

### Enzyme assay

BVMO activity was measured by monitoring the decrease in absorbance of NADPH at 340 nm (ε_NADPH340_ = 6.22 cm^−1^ mM^−1^) after addition of the substrate. Exceptions were made for 4′-hydroxyacetophenone that strongly absorbs at 340 nm and for which NADPH depletion was measured at 370 nm (ε_NADPH370_ = 2.7 cm^−1^ mM^−1^), and ethionamide for which the product formation was measured at 400 nm (ε_ETH400_ = 1.0 cm^−1^ mM^−1^) instead of cofactor depletion. All spectrophotometric measurements were made using UV-1650 PC Spectrophotometer (Shimadzu, Kyoto, Japan) at 25°C, unless otherwise mentioned. The reactions were done in 50 mM sodium phosphate buffer pH 7.5, containing 10 mM KCl, 60 μM NADPH and 5 mM substrate (except steroids which were used at 0.5 mM due to their low solubility). The final enzyme concentration in the assay was 0.024 mg/ml. Steady state kinetics were measured by varying either the cofactor NADPH or the substrate (phenylacetone or 2-methylcyclohexanone). The kinetic parameters (*K*_m_ and *k*_cat_) of the BVMO were determined using the Lineweaver-Burk plot of the Michaelis-Menten equation under steady-state conditions.

### Effect of pH and temperature on BVMO4 activity and stability

Effects of pH and temperature on the enzyme activity were determined by assaying the enzyme at different pH (pH 5–9) and temperatures (10–45°C). To determine the effect of pH on the enzyme stability, the enzyme was kept in the buffers at 4°C for 24 days and the residual activity was determined. Similarly, thermal stability of BVMO4 was studied by incubating the enzyme at 35°C in 50 mM sodium phosphate buffer, pH 7.5 and measuring the residual activity of samples withdrawn periodically. The enzyme solutions used to determine the enzyme stability was 0.5 mg/ml.

### Effect of freezing-thawing on BVMO4 activity

The enzyme solution (0.5 mg/ml) was subjected to freeze-thaw cycles in the presence of various cryoprotectants, added at a final concentration: glycerol 20% (v/v), sorbitol 1.5 M, trehalose 0.5 M, betaine 1.5 M, DMSO 20% (v/v), 2-methyl-2,4-pentanediol 20% (v/v), PEG 600 20% (w/v), BSA 20% (w/v), ectoine 0.5 M, hydroxyectoine 0.5 M. The enzymatic activity was measured (using phenylacetone as substrate) before freezing and after freeze-thaw cycles.

### BVMO4 activity in the presence of salt and organic solvents

To determine salt tolerance, the enzyme activity on phenylacetone was measured at pH 7.5 in the presence of 0-1.2 M NaCl. Similarly, the effect of organic solvents on the activity of the enzyme was determined by measuring the activity in the presence of 20% (v/v) organic solvents.

### Nucleic acid sequences

The nucleic acid and protein sequences reported in this work are available at GenBank under the deposition number KF319017.

## Results

### Gene and protein sequence analysis

**Figure 1:**
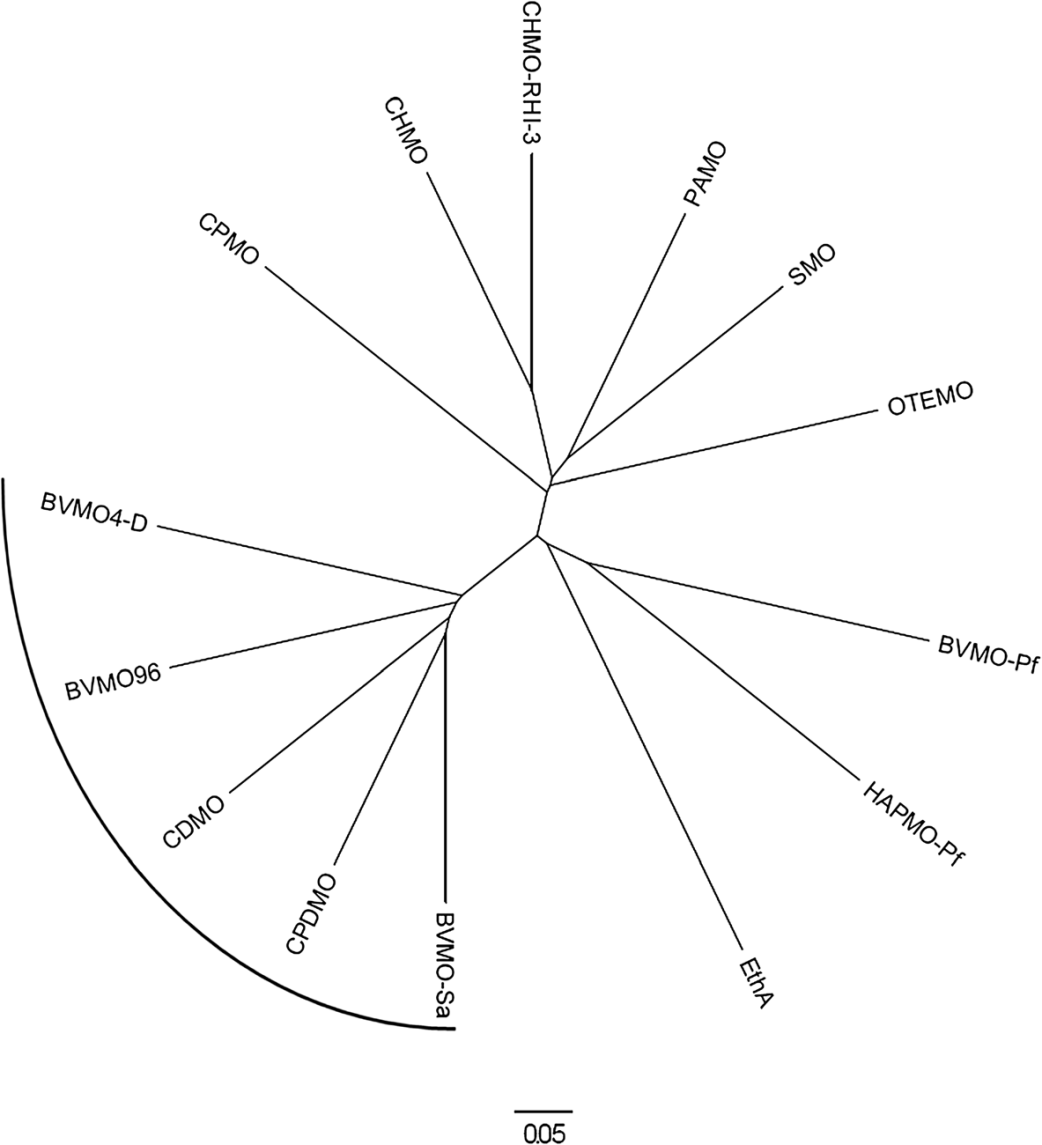
**A phylogenetic tree of BVMOs.** The alignment was done using T-Coffee and the phylogenetic tree was derived using Clustal W2. The BVMOs represented on the tree are: BVMO4 *Dietzia* sp. D5 (AGY78320.1); BVMO96 *Streptomyces coelicolor* A3(2) (NP_624628.1); CDMO *Rhodococcus ruber* SC1 (AAL14233.1); CPDMO *Pseudomonas* sp. HI-70 (BAE93346.1); BVMO-Sa is BVMO *Streptomyces avermitilis* MA-4680 (NP_824170.1); EthA is EthA3854c *Mycobacterium tuberculosis* H37Rv (NP_218371.1); HAPMO-Pf is HAPMO *Pseudomonas fluorescens* ACB (Q93TJ5.1); BVMO-Pf is BVMO *Pseudomonas fluorescens* DSM50106 (AAC36351.2); OTEMO *Pseudomonas putida* (3UOV_A); SMO *Rhodococcus rhodochrous* (BAF48129.1); PAMO *Thermobifida fusca* YX (YP_289549.1); CHMO-RHI-3 is CHMO *Rhodococcus* sp. HI-31 (3UCL_A); CHMO *Acinetobacter* sp. NCIMB9871 (BAA86293.1); CPMO *Comamonas* sp. NCIMB9872 (Q8GAW0.3).

The gene encoding BVMO4 was amplified from the genomic DNA of *Dietzia* sp. D5. The enzyme is composed of 612 amino acids, which makes it larger than most BVMOs which are 500 to 550 amino acids long. BLAST search against database sequences revealed highest identity (63%) with the unstudied putative BVMO from *Gordonia terrae* NBRC 100016 (ZP_09801205), while among characterized BVMOs, 41–44% sequence similarity was observed with a group of BVMOs whose most studied member is CPDMO and that like BVMO4 are about 600 amino acids long in their primary structure. A phylogenetic tree of BVMO4, CPDMO, CPDMO-like and other known BVMOs is shown in Figure 1.

Comparison of the DNA sequence of the genome fragment of *Dietzia* sp. D5 containing BVMO4 gene with the recently published genome of a closely related organism, *Dietzia cinnamea* P4 (Procópio et al. [2012]) showed that most of the genes neighboring the BVMO gene are present in the two organisms and have the same order, although two of the predicted ORFs in *Dietzia* sp. D5 are missing in *D. cinnamea* P4. The missing ORFs are the ones encoding BVMO4 and a TetR transcriptional regulator gene.

### Expression and purification of BVMO4

**Figure 2:**
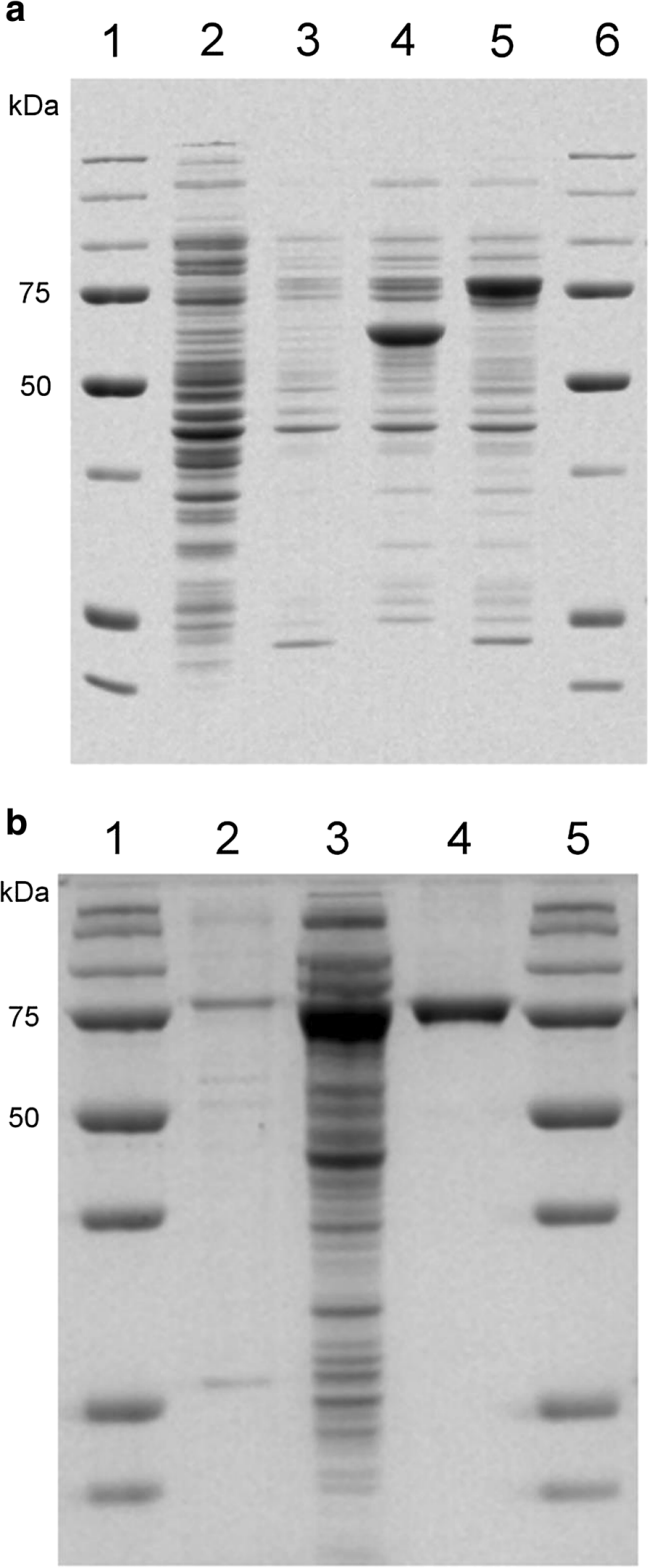
**Expression and purification of BVMO4. a)** Soluble crude extracts of different *E. coli* strains expressing BVMO4. Lane 1 and 6, protein marker (AllBlue, BioRad); lane 2, *E. coli* BL21(DE3); lane 3, *E. coli* Rosetta(DE3); lane 4, *E. coli* ArcticExpress(DE3)-RP; and lane 5, *E. coli* BL21-CodonPlus(DE3)-RP. **b)** Purified BVMO4. Lane 1 and 5, protein marker (AllBlue, BioRad); lane 2, cell debris; lane 3, soluble cell extract; lane 4, purified protein after His-tag affinity chromatography.

*E. coli* strains BL21(DE3), Rosetta(DE3), ArcticExpress(DE3)-RP and BL21-CodonPlus(DE3)-RP were used to evaluate the expression of BVMO4 in soluble form. The best result was achieved with *E. coli* BL21-CodonPlus(DE3)-RP and SDS-PAGE of the crude cell extract shows a prominent band at about 75 kDa (Figure 2a) which is close to the *in silico* predicted mass of 72 kDa. *E. coli* BL21(DE3) cells were unable to express BVMO4 while *E. coli* Rosetta(DE3) and ArcticExpress(DE3)-RP resulted in modest levels of expression; however, the cell growth was considerably lower when compared to BL21-CodonPlus(DE3)-RP. Thus, BL21-CodonPlus(DE3)-RP was chosen as the expression host. In the case of *E. coli* ArcticExpress(DE3)-RP the thick protein band at approximately 55 kDa in Figure 2a is a chaperone that is expressed to help the folding of the BVMO.

The enzyme was purified by His-tag affinity chromatography and the summary of the enzyme purification steps is given in Table 1. The enzyme lost its activity during the purification process but was recovered upon addition of FAD. The specific activity of the pure enzyme was about 0.65 U/mg protein when assayed using phenylacetone as substrate.

**Table 1:** Summary of the recombinant BVMO4 purification steps

**Steps**	**Total protein (mg)**	**Total activity (U)**	**Specific activity (U/mg)**	**Purification fold**
Crude extract	78.86	2.95	0.037	1
Purified enzyme	4.95	3.39	0.648	17.5

### Effect of pH and temperature on activity and stability of BVMO4

**Figure 3:**
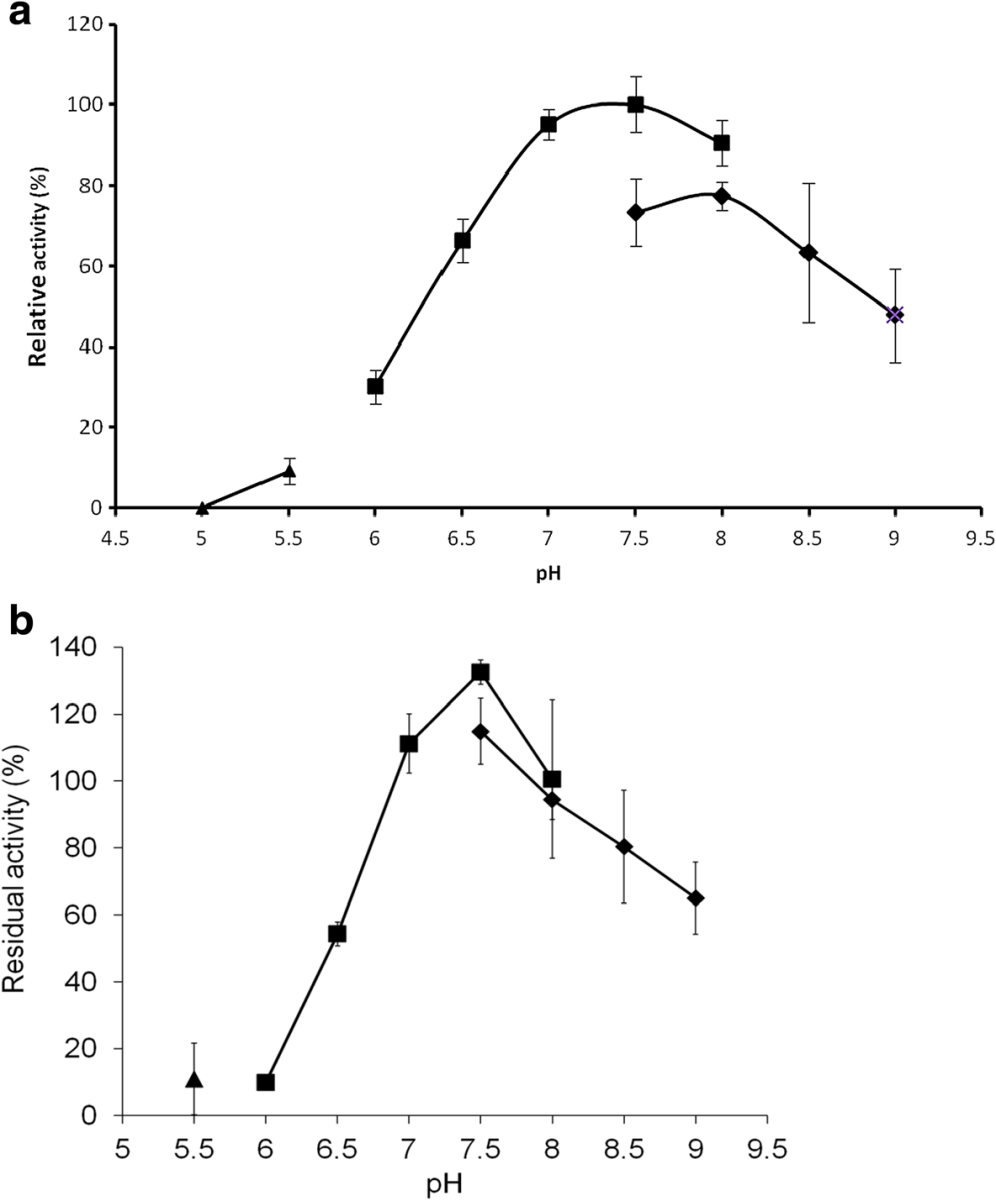
**Effect of pH on activity and stability of BVMO4. a)** Effect of pH on the activity of BVMO4 was determined by assaying the enzyme at 25°C in 50 mM sodium acetate buffer (▲) pH 5 to 5.5, sodium phosphate buffer (■) pH 6 to 8, and Tris–HCl (♦) pH 7.5 to 9. **b)** The residual activity of BVMO4 after 24 days at 4 °C and different pH values was measured at 25°C in 50 mM sodium acetate (▲), sodium phosphate (■) and Tris–HCl (♦) buffers.

Effect of pH on the activity of the pure enzyme was determined by performing the assay in a pH range from 5 to 9. The enzyme was optimally active at pH 7.5 and exhibited more than 50% of its optimal activity between pH 6.5 and 9.0 (Figure 3a). When stored at 4°C, the enzyme displayed highest stability in a pH range of 7.0–8.0 (Figure 3b).

**Figure 4:**
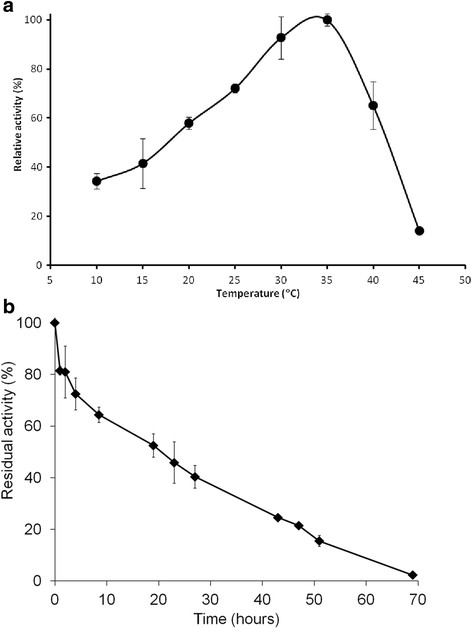
**Effect of temperature on activity and stability of BVMO4. a)** Effect of temperature on the activity of BVMO4 was determined by assaying the enzyme at different temperatures in 50 mM sodium phosphate buffer, pH 7.5. **b)** The BVMO4 stability was studied by incubating the enzyme at 35°C in 50 mM sodium phosphate buffer, pH 7.5. Samples were taken periodically and the residual enzyme activity was determined.

The enzyme was optimally active at 35°C (Figure 4a) and retained about 50% and 20% of its initial activity after 20 h and 48 h of incubation at 35°C, respectively (Figure 4b). The recombinant BVMO can be stored frozen at −20°C for several months without loss of activity. Moreover frozen aliquots of the enzyme proved to be resistant to at least three freeze-thaw cycles both in the presence and absence of cryoprotectants. However, the enzyme lost most of its activity when 2-methyl-2,4-pentanediol was used as cryoprotectant (data not shown).

### Determination of BVMO4 substrate scope

A total of 28 compounds were studied as substrates for BVMO4 (Table 2). Activity on phenylacetone was used as a reference (100%) to determine the relative activity of the enzyme on these different substrates. The highest activity was measured for 2- and 3-methylcyclohexanone, phenylacetone, bicyclo[3.2.0]hept-2-en-6-one and menthone. It was noted that the position of the methyl substitution in the series of methylcyclohexanones influenced the enzyme activity: 2-methylcyclohexanone had the higher relative activity (155%), 3-methylcyclohexanone was a moderately good substrate (90% relative activity) and 4-methylcyclohexanone reacted poorly (15% relative activity). Among aliyclic ketones, the highest activity was achieved when cyclohexanone was used as substrate, though they do not appear to be among the best substrates for BVMO4. Aliphatic ketones show approximately the same reactivity as cyclohexanone regardless of the chain length. The enzyme oxidized phenylacetone readily but there was little or no detectable activity when the substrate was a related structure, such as acetophenone and 4′-hydroxyacetophenone, which indicates the high selectivity of BVMO4 among these substrates. In addition, there was no detectable activity on benzaldehyde. Its activity on sulfides and ketosteroids, was moderate, and the oxidation on the D ring of ketosteroids seems preferred. A detailed study on the activity and enantioselectivity of BVMO4 with sulfide and aldehyde substrates has been reported elsewhere (Bisagni et al. [2014]).

**Table 2:** Relative activity of BVMO4 towards different substrates

**Substrates**	**Relative activity %**
**Alicyclic ketones**	
Cyclobutanone	20.2 ± 6.1
Cyclopentanone	12.7 ± 5.9
Cyclohexanone	22.0 ± 3.5
Cycloheptanone	12.0 ± 3.6
Cyclooctanone	9.3 ± 3.9
Cyclopentadecanone	0.0
**Substituted alicyclic ketones**	
2-methylcyclohexanone	155.3 ± 24.8
3-methylcyclohexanone	90.5 ± 1.9
4-methylcyclohexanone	14.6 ± 0.7
2-cyclohexen-1-one	5.4 ± 1.1
**Bicyclic ketones**	
Bicyclo[3.2.0]hept-2-en-6-one	103.0 ± 6.4
Norcamphor	6.3 ± 2.2
Beta tetralone	0.0
Alpha tetralone	10.8 ± 2.7
**Aliphatic ketones**	
2-heptanone	20.7 ± 0.7
2-octanone	22.6 ± 1.7
2-decanone	22.0 ± 0.4
2-pentadecanone	20.9 ± 3.5
**Aliphatic ketone with aromatic substituents**	
Phenylacetone	100.0 ± 6.2
Acetophenone	2.4 ± 0.8
4′-hydroxyacetophenone	0.0
Benzaldehyde	0.0
**Sulfides**	
Thioanisole	33.0 ± 13.5
Ethionamide	32.0 ± 1.8
**Other ketones**	
Progesterone^a^	23.0 ± 0.6
Estrone^a^	17.0 ± 0.4
Testosterone^a^	6.6 ± 2.4
Menthone	77.1 ± 2.7

The activity on phenylacetone which is considered as 100% was 1.87 mU. The initial substrate concentration was 5 mM. ^a^Substrate concentration was 0.5 mM.

Steady state kinetic properties of BVMO4 were determined for the cofactor NADPH and two of the best substrates, 2-methylcyclohexanone and phenylacetone (Table 3). The enzyme displayed relatively higher affinity (lower *K*_m_) for 2-methylcyclohexanone but higher *V*_max_ and *k*_cat_ for phenylacetone as a result of which *k*_cat_/*K*_m_ were similar for the two substrates. The *K*_m_ for NADPH was about 10 μM while there was no detectable activity when NADH was used as a cofactor, which indicates that BVMO4 is strictly NADPH dependent*.*

**Table 3:** Steady state kinetic properties of BVMO4

	***K***_**m**_**(mM)**	***V***_**max**_**(mM s**^**−1**^**)**	***k***_**cat**_**(s**^**−1**^**)**	***k***_**cat**_**/*K***_**m**_**(mM**^**−1**^**s**^**−1**^**)**
**Phenylacetone**	0.829 ± 0.163	7.90E-05 ± 2.31E-06	0.634 ± 0.104	0.77 ± 0.03
**2-methylcyclohexanone**	0.507 ± 0.142	5.11E-05 ± 1.31E-05	0.370 ± 0.095	0.73 ± 0.02
**NADPH**	0.011 ± 0.002	7.00E-05 ± 7.80E-06	0.507 ± 0.057	45.06 ± 1.52

### Enzyme activity in presence of sodium chloride and organic solvents

**Figure 5:**
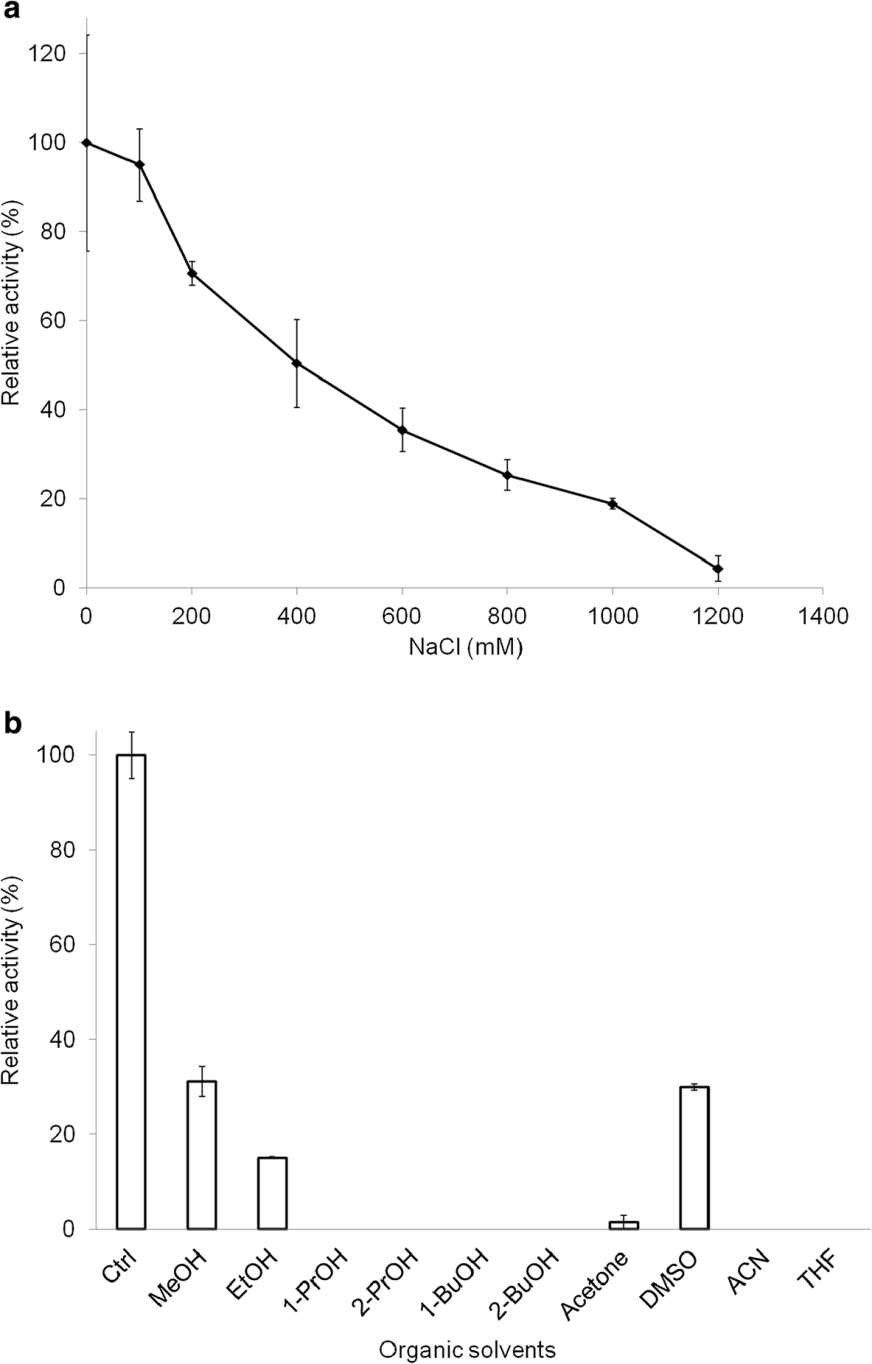
**Effect of NaCl and organic solvents on the activity of BVMO4. a)** Activity of BVMO4 in the presence of varying concentration of NaCl. The assay was performed at 25 °C in 50 mM sodium phosphate buffer, pH 7.5. **b)** Effect of different organic solvents (20% v/v) on the activity of BVMO4.

*Dietzia* sp. D5, the source organism for BVMO4, as well as other *Dietzia* spp., are halotolerant (Plakunov et al. [2008]) and hence the salt tolerance of the enzyme was investigated. At 0.4 M NaCl, the enzyme exhibited more than 50% of the activity in the absence of salt, and the activity decreased steadily with increasing salt concentration up to 1.2 M, at which there was no detectable activity (Figure 5a).

The influence of organic solvents frequently used in organic synthesis, on BVMO4 activity was also investigated. The solvents were added to the reaction mixture at a final concentration of 20% (v/v). Some activity was detected in the presence of methanol, DMSO and to a lesser extent ethanol but there was no detectable activity in the other solvents tested (Figure 5b).

## Discussion

The sequence similarity analysis of BVMO4 revealed that it is related to a group of Baeyer-Villiger monooxygenases which are characterized by longer amino acid sequences compared to most other BVMOs. The most studied member of this group of BVMOs is CPDMO, a BVMO from *Pseudomonas* sp. HI-70 (Iwaki et al. [2006]; Beneventi et al. [2009]; Fink et al. [2011]). The other BVMOs belonging to this group are both of microbial and fungal origin (Miyamoto et al. [1995]; Jiang et al. [2009]; Qiao et al. [2011]) and are known to be very versatile in terms of their substrate scope, and efficiently catalyze the oxidation of a wide range of substrates, which indicates a broader application potential (Fink et al. [2012]; Bianchi et al. [2013]).

*Dietzia* spp. genomes have high GC content; in fact the BVMO4 encoding gene has over 67% GC content and a number of rare codons. Expression of high GC genes containing multiple rare codons in *E. coli* is difficult and most of the commonly used strains such as *E. coli* BL21(DE3) cannot properly express such genes. However, some *E. coli* strains developed for the expression of this kind of genes are appearing in the market. Among these strains are *E. coli* Rosetta(DE3), BL21-CodonPlus(DE3)-RP and ArcticExpress(DE3)-RP which successfully expressed BVMO4 gene as shown in Figure 2a while expression in BL21(DE3) was not possible. BL21-CodonPlus(DE3)-RP was better than the other two strains; protein expression is much higher and the culture has grown to cell density comparable to that of BL21(DE3), while the other strains, especially Rosetta(DE3), have a much lower growth rate.

The recombinant BVMO4 expressed in *E. coli* BL21-CodonPlus(DE3)-RP cells was purified to homogeneity by immobilized metal ion affinity chromatography. It was observed that the enzyme completely lost its activity during the purification process. Since the activity of the purified enzyme was recovered with addition of FAD, the loss of activity during purification is believed to be due to loss of the cofactor and such phenomenon has been reported before (Nam et al. [2002], Malito et al. [2004]). Addition of FAD after the enzyme purification not only restored the activity but also resulted in a higher level of total activity in the purified enzyme than the crude form (Table 1). This might be due to an insufficient production of FAD by the *E. coli* cells, which may result in a part of the expressed enzyme in the crude extract being inactive due to lack of the cofactor. The addition of FAD precursors such as riboflavin, to the reaction media might increase the enzymatic activity in the crude extract (Yoshikane et al. [2004]; Wang and Wang [2007]).

The optimal pH for the activity of BVMO4 (pH 7.5) is slightly lower than the pH optima of most BVMOs that lies between pH 8 and 9.5. However, it is similar to that of cyclopentanone monooxygenase (pH 7.7) from a *Pseudomonas* strain (Griffin and Trudgill [1976]). BVMO4 is less active and less stable in acidic solution which has also been observed for other BVMOs (Secundo et al. [2005]; Völker et al. [2008]; Rehdorf et al. [2009]).

Although several BVMOs are available as recombinant enzymes, they have so far been very seldom used in industrial applications (Baldwin et al. [2008]). Poor stability is one of the factors that hindered their applications (Clouthier and Pelletier [2012]). A number of purified monooxygenases rapidly lose activity even when stored at 4°C or in a frozen state (Völker et al. [2008]; Kadow et al. [2012]). A CHMO from *Acinetobacter*, one of the most studied BVMOs, has a half-life of 24 hours at 25°C (Zambianchi et al. [2002]) and a similar property was observed for a HAPMO (Rehdorf et al. [2009]). Another enzyme, OTEMO is totally inactive after 4 hours at 25°C and loses half of its initial activity within 24 hours at 4°C (Kadow et al. [2012]). Interestingly, BVMO4 displayed higher stability than the great majority of BVMOs. Moreover, BVMO4 can be stored and freeze-thawed repeatedly without significant loss of activity.

As applications of BVMOs involve oxidation of organic compounds, it is important to know the effect of organic solvents, such as those used to dissolve substrates, on the enzyme activity. The activity of BVMO4 in the presence of organic solvents at 20% (v/v) final concentration was comparable to what has been reported for a CHMO (Secundo et al. [2011]) but lower than a PAMO (de Gonzalo et al. [2006,2012]). Similarily, high salt concentration can affect enzyme hydration and enzymes that are stable at high salt concentrations are preferred for industrial applications (Woodley [2013]). BVMO4 retained more than 50% of its salt-free activity at NaCl concentration of up to 0.4 M. Although this concentration does not seem outstandingly high, the enzyme has shown moderate resistance. Such data is however not available for other BVMOs.

BVMO4 has a wide substrate scope and oxidizes substrates with aromatic moiety, substituted cyclic ketones and ketones in multi-ring compounds. In addition, the enzyme oxidized alicyclic and linear aliphatic ketones, thiols and bulky substrates, such as steroids, although at a lower rate. Despite the primary sequence of the enzyme is close to BVMOs belonging to CPDMO group, it poorly oxidizes medium-sized ketones and did not show activity on cyclopentadecanone. Some discrepancies in the enzyme sequence can be the cause for the different affinity for bulky alicyclic ketones. Thus, it can be speculated that BVMO4 is a novel enzyme. In fact, when compared to other BVMOs in the CPDMO group, this enzyme branches out early in the phylogenetic tree (Figure 1). When the kinetic parameters were measured with phenylacetone and 2-methylcyclohexanone as substrates, the *K*_m_ values were in the order of hundreds of μM (Table 3). The *K*_m_ of cyclohexanone for CHMO (Trudgill [1990]) is two orders of magnitude lower than the ones measured for BVMO4, indicating that although phenylacetone and 2-methylcyclohexanone are readily oxidized, BVMO4 has relatively low affinity for the substrates.

The results of this study show that BVMO4 is an interesting enzyme to catalyze oxidation of various substrates. In particular, its wide substrate scope and high stability make this enzyme a potential candidate for various Baeyer-Villiger oxidations. Further work to demonstrate its potential on biotransformations of different substrates, understanding its structure-function relationship and improving its property through mutagenesis is currently being considered.

## Competing interests

The authors declare that they have no competing interests.
